# Endoglin (CD105) overexpression is associated with an immunosuppressive tumor microenvironment in murine lung cancer models and human lung adenocarcinoma

**DOI:** 10.1038/s41598-026-55711-6

**Published:** 2026-06-12

**Authors:** Claudia Ollauri-Ibáñez, Blanca Ayuso-Íñigo, Inés Solano-SC, Paula Díez, Martín Perez-Andres, José M. Muñoz-Félix, Alicia Rodríguez-Barbero, Miguel Pericacho

**Affiliations:** 1 Instituto de Investigación Biomédica de Salamanca (IBSAL), Salamanca, 37007 Spain; 2 Facultad de Medicina, Departamento de Fisiología y Farmacología, Universidad de Salamanca, Salamanca, 37007 Spain; 3 Facultad de Ciencias de la Salud, Departamento de Bioquímica, Biología Molecular y Fisiología, Universidad de Valladolid, Soria, 42004 Spain; 4 Translational and Clinical Research Program, Cancer Research Center (IBMCC, CSIC – University of Salamanca), Salamanca, Spain; 5 Cytometry Service, Department of Medicine, NUCLEUS, University of Salamanca, Salamanca, Spain; 6 Facultad de Biología, Departamento de Bioquímica y Biología Molecular, Universidad de Salamanca, Salamanca, 37007 Spain; 7 Facultad de Medicina y Ciencias de la Salud, Departamento de Biología Funcional, Área de inmunología, Universidad de Oviedo, 33006 Oviedo, Spain

**Keywords:** Angiogenesis, Tumor microenvironment (TME), Cold tumor, Endoglin (CD105), Lung cancer, Cancer models, Tumour angiogenesis, Tumour immunology

## Abstract

The tumor microenvironment (TME) is a major determinant of tumor response to different therapies, especially immunotherapy. Tumors with high CD8^+^ T-cell infiltration that respond well to immunotherapy are referred to as “hot” tumors, whereas “cold” tumors, with an immunosuppressive microenvironment, respond less effectively to this therapy. Endoglin (CD105) plays a critical role in angiogenesis, and its overexpression has been associated with a poorer prognosis in several types of cancer. This study aims to investigate whether high endoglin levels are associated with a cold microenvironment in tumors. Transgenic mice ubiquitously overexpressing human endoglin (*ENG*^+^) and wild-type C57BL/6J mice (WT) were used to analyze the TME in a Lewis Lung Carcinoma (LLC) subcutaneous xenograft model and in a lung cancer model. Tumors developed in *ENG*^+^ mice exhibited increased hypoxia, reduced CD8^+^ T-cell infiltration and an increased presence of immunosuppressive cells, such as M2 TAMs and Treg, compared to WT tumors. Thus, these tumors can be categorized as cold tumors. In addition, the analysis of the TME and endoglin expression in human lung adenocarcinoma samples showed that cold tumors have higher endoglin levels than hot tumors. These findings suggest that a hypoxic and immunosuppressive microenvironment may contribute to the poorer prognosis of tumors with high levels of this protein. This study highlights the potential of endoglin as a marker to predict the response to immunotherapy and to guide personalized treatment strategies in cancer patients.

**Supplementary Information:**

The online version contains supplementary material available at 10.1038/s41598-026-55711-6.

## Introduction

Antiangiogenic drugs became one of the great promises of cancer treatment when, in the 1970s, Folkman described neoangiogenesis as a critical process for the development of solid tumors^1^. Promising results from preclinical studies have led to their routine use in patients with various malignancies, including lung and renal cell cancer, among others. However, it has been demonstrated that they do not show the expected success in patients^2,3^. Instead, the lack of blood vessels creates a hypoxic environment that favors the proliferation of the most resistant and aggressive tumor cells^4,5^. In 2018, Dr. Alison and Dr. Honjo were awarded the Nobel Prize in Medicine for their research on immunotherapy^6^. This type of therapy harnesses and enhances the ability of immune system effector cells to selectively destroy malignant cells while preserving healthy tissue^7^. Immunotherapy is already a first-line treatment for non-small cell lung cancer^8–11^, as well as other solid tumors, but unfortunately, the objective response rate is only between 10% and 40%^8,10^. It has been shown that the response to these drugs depends largely on the nature of the tumor microenvironment (TME)^12^.

The TME is formed by malignant and normal cells that constitute a tumor and their interactions through cytokines, chemokines, growth factors and enzymes that remodel the extracellular matrix^13^. Depending on the composition of the TME, there are two types of tumors: hot tumors, which respond well to immunotherapy, and cold tumors, which do not respond to this treatment. Hot or inflamed tumors are characterized by a TME with highly infiltrating anti-tumor cells, such as CD8^+^ T lymphocytes or M1 tumor-associated macrophages (TAMs), and low numbers of immunosuppressive cells, such as myeloid-derived suppressor cells (MDSCs), M2 TAMs or regulatory T lymphocytes (Tregs). In contrast, cold or non-inflamed tumors contain high numbers of tumor-promoting cells and low infiltration of anti-tumor immune cells^12,14^. Furthermore, these cold tumors are associated with poor vascular function, leading to poor perfusion and favoring the development of hypoxia, which promotes the survival of the most aggressive cells and the recruitment of anti-inflammatory cells^4,12,15–17^. For these reasons, identifying markers that can reveal the nature of the tumor and predict whether a patient will respond well or poorly to immunotherapy, from a simple biopsy, will prevent patients from undergoing ineffective treatments, avoiding possible side effects and bringing us closer to personalized medicine.

One of these potential markers may be endoglin (CD105), a transforming growth factor-β (TGF-β) coreceptor that plays a critical role in various pathophysiological processes, including angiogenesis^18,19^, whose overexpression has been associated with poorer prognosis in different types of cancer^20,21^. Our group recently showed that endoglin overexpression preserves an active phenotype in endothelial cells, thereby preventing vascular maturation. In the case of tumors, this does not lead to increased vascularization or tumor mass growth, as expected, but rather to more vascular lacunae and hemorrhages. Moreover, mice overexpressing endoglin (*ENG*^*+*^) have more circulating tumor cells and develop more lung metastases, which may be responsible for the poorer prognosis of tumors with high endoglin levels^22^.

Therefore, the aim of this work is to analyze whether elevated endoglin levels are associated with cold tumors. For this purpose, we will use the hEng transgenic murine model (*ENG*^*+*^) to investigate whether endoglin overexpression is correlated with more hypoxic tumors and an immunosuppressive TME, which, together with the alterations in angiogenesis already described, define tumors as cold. In addition, we will determine whether elevated endoglin expression is also associated with cold tumors in human lung adenocarcinoma samples.

## Materials and methods

### Mice

All animal procedures were conducted in strict compliance with the European Community Council Directive (2010/63/EU) and Spanish legislation (RD1201/2005 and RD53/2013). The protocols were approved by the University of Salamanca Ethical Committee (Permit number #305). The study is reported in accordance with ARRIVE guidelines (https://arriveguidelines.org). Transgenic mice ubiquitously overexpressing endoglin (*ENG*^*+*^) were generated by the previous group leader via microinjection of a pCAGGS vector containing the complementary DNA sequence (cDNA) of human endoglin in the fertilized eggs of CBAxC57BL/6J mice, as previously described^22,23^ and are bred in the university animal facility. Wild-type C57BL/6J mice (WT) were used as controls. Animal selection was genotype-based, and no randomization or blinding was performed. Animals were housed under specific pathogen-free conditions at the University of Salamanca facilities in a temperature-controlled room with a 12-h light/dark cycle and reared on standard chow and water provided ad libitum. A similar number of 8–13-week-old male and female *ENG*^*+*^ and WT mice were used for the experiments. For animal anesthesia, 2% isoflurane in oxygen was used, and heat was provided during recovery. Animals were sacrificed via CO_2_ inhalation or cervical dislocation, depending on the experiment requirements.

### Human samples

Access to human samples was granted by the Ethics Committee of the University of Salamanca (protocol code 437) within the legal framework of informed consent (Law 41/2002), the practice of biomedical research (Law 14/2007) and data processing and privacy in the national (Organic Law 3/2018) and European (2016/679/EU) context. Written informed consent was obtained from all donors for donation of samples to the biobank and their use in biomedical research. All methods involving human samples were performed in accordance with the Declaration of Helsinki and with all relevant guidelines and regulations.

Fifty human samples of lung adenocarcinoma (LUAD) were provided by Red de Biobancos de Castilla y León (BEOCyL) as formalin-fixed paraffin-embedded (FFPE) blocks of tissue. All the samples were classified according to their histopathological pattern according to the WHO classification of 2021 by hematoxylin/eosin (H&E) staining. This classification was based on the analysis of 2 independent investigators under the supervision of a pathologist from the Hospital Universitario de Salamanca. Their vascular pattern was also analyzed and classified as angiogenic or non-angiogenic by immunohistochemistry with CD31 as an endothelial marker, and Weigert-Van Gieson staining to visualize the collagen and elastin fibers. The fifteen samples that were classified as solid adenocarcinomas and presented an angiogenic vascular pattern were selected for further analyses.

### Cell culture

Lewis Lung Carcinoma (LLC) cells, provided by Dr. Aliño (Department of Pharmacology, University of Valencia, Spain), were cultured under adherent conditions in Dulbecco’s modified Eagle’s medium (DMEM) (Thermo Fisher Scientific) supplemented with 10% fetal bovine serum (FBS) (Thermo Fisher Scientific) and 50 U/mL penicillin–streptomycin (Thermo Fisher Scientific) and maintained in a 90% RH, 5% CO_2_ atmosphere at 37 °C.

### LLC cell subcutaneous xenograft model

The subcutaneous xenograft model was performed as previously described^22^. Briefly, 10^6^ LLC cells were resuspended in 50 µL of Matrigel^®^ (Corning) and subcutaneously inoculated into the flanks of anesthetized mice. Two different tumors were induced in the dorsum of each mouse, with an initial diameter of 2–3 mm resulting from the cell–Matrigel^®^ mixture. Ten days later, the mice were humanely euthanized via CO_2_ inhalation. The tumors were carefully removed and frozen in liquid nitrogen, fixed in 4% paraformaldehyde (PFA) or digested in collagenase, depending on the subsequent analysis.

### LLC cell lung model

The lung tumor model was performed as previously described^24^. Briefly, 10^5^ LLC cells were resuspended in 100 µL of PBS and injected intravenously into anesthetized mice. Previously, the mice were prewarmed for 10 min via a heating pad to induce vasodilatation. Fifteen days after tumor cell inoculation, the mice were humanely euthanized via cervical dislocation. Then, the trachea was cannulated, and the lungs were filled with 4% PFA to maintain tissue structure. The lungs were subsequently removed from the thoracic cavity and fixed in 4% PFA for 24 h. The lung lobes were dissected before being embedded in paraffin for histological analysis.

### Flow cytometry

Half of the subcutaneous tumor was digested in 0.5 mg/mL collagenase from *Clostridium histolyticum* (Sigma‒Aldrich) and 0.02 mg/mL deoxyribonuclease I from bovine pancreas (Sigma‒Aldrich) for 1.45 h at 37 °C under agitation. The digestion mixture was then passed through a 70 μm-pore Falcon^®^ cell strainer (Corning) and washed three times with PBS. The cell pellet was labeled with the antibody cocktail (Table S1) in the dark at room temperature for 15 min. After two washes with PBS, 10^6^ cells were acquired on a BD FACSCanto™ II (BD Biosciences). Data were analyzed via Infinicyt 1.7 (Cytognos). Gating strategy and flow plots are shown in Figure S1 and the combination of markers used to identify each cell type is indicated in Table S2.

### Histological analysis: Immunofluorescence and immunohistochemistry

Both subcutaneous and lung tumors destined for histological analysis were fixed in 4% PFA for 24 h. The samples were subsequently embedded in paraffin in the Comparative Molecular Pathology Unit of the Cancer Research Centre of Salamanca. Human samples were provided as formalin-fixed paraffin-embedded (FFPE) blocks of tissue.

For immunohistochemistry, 2 μm sections were deparaffinized with xylene and rehydrated in a decreasing gradient of ethanol (100–70%). Antigens were unmasked with 10 mM sodium citrate buffer. The sections were incubated overnight at 4 °C with the primary antibody (Table S3) and for 1 h at room temperature with the secondary antibodies (Table S4). Following, they were incubated with the DAB Substrate Kit (Abcam ab64238) for up to 10 min. Finally, the nuclei were counterstained with hematoxylin, and the sections were dehydrated and mounted with DPX mounting medium (Casa Álvarez 10-8500). Images were acquired using an Olympus BX51 optical microscope and a PANNORAMIC Scan (3DHISTECH’s).

For immunofluorescence, antigens from 2-µm sections were also unmasked with 10 mM sodium citrate buffer. The sections were incubated overnight at 4 °C with the primary antibody (Table S3) and in the dark for 1 h at room temperature with the secondary antibodies (Table S4). The cell nuclei were stained by incubating for 5 min with Hoechst before they were mounted with Prolong^®^ Gold Antifade (Thermo Fisher Scientific). Images were acquired using an Axiovert 200 M fluorescence microscope (Nikon) and a Nanozoomer S60 (Hamamatsu).

### Image quantification

Researchers who manually analyzed the images were blinded to the genotype of the samples.

### Subcutaneous xenograft model

Ten random areas were selected at 20x magnification. Within each area, two subareas were marked around the blood vessels at a distance of three nuclei from the vessels (perivascular area). Then, within each subarea, vascular density and mural coverage were analyzed. For this purpose, the number of total blood vessels was quantified (based on the vessel marker Endomucin) with respect to the tumor area (in pixels), and the coverage of each vessel was also evaluated by assigning a score for the mural cell marker α-SMA (1 for completely mature vessels/high coverage, 0.5 for partially mature vessels, and 0 for immature vessels/low coverage). The final coverage percentage was then calculated as the ratio of the α-SMA score relative to the number of vessels in each area. Furthermore, to analyze perivascular lymphocyte infiltration, the number of CD3-positive cells was counted relative to the perivascular area (µm^2^). To analyze hypoxia, the areas positive for Glut1 or CaIX were selected with respect to the total tumor area (%).

For cell infiltration analysis, several random areas were selected at 20x magnification, and the number of positive cells for CD8a, FoxP3, CD206 or Arg1 was counted.

### Lung model

Tumors were fully photographed at 10x or 20x magnification, depending on the marker, and the entire tumor area was analyzed using Photoshop CS6 (in pixels). First, in each tumor, the angiogenic area and/or non-angiogenic area were selected. Tumors that presented at least 35% of the angiogenic area with respect to the total tumor area were considered angiogenic tumors. The remaining histological analyses were performed on the tumors identified as angiogenic, as in the subcutaneous model.

### Human lung adenocarcinoma

Only samples previously classified as solid (growth pattern) and angiogenic (vascular pattern) were used in this study. The levels of endoglin expression were evaluated by assigning a microvascular density (MVD) score (0 for no staining, 1 for low expression and staining, 2 for medium expression, and 3 for high expression). For TME characterization, several random areas were selected, and the number of cells positive for CD8 or FoxP3 was counted. M2 TAMs infiltration was evaluated by measuring positive areas for CD206 with respect to the total tumor area (%). Tumors were classified as cold when CD8^+^ cell infiltration was low (CD8^low^) and hot when CD8^high^. For medium infiltration of CD8^+^ cells, Tregs and M2 TAMs were also considered: hot for low FoxP3 and/or CD206, and cold for high FoxP3 and/or CD206 (Fig. 6J).

### Cytokine gene expression (RT‒PCR)

RNA was isolated from frozen tissue using NucleoSpin^®^ RNA (Macherey-Nagel), and cDNA synthesis was subsequently performed with iScript RT Supermix (Bio-Rad) according to the manufacturer’s instructions. For qPCR, Supermix iQTM SYBR^®^ Green (Bio-Rad) and an iQTM 5 System (Bio-Rad) were used. Gene expression results were normalized to ribosomal protein S13 (*Rps13*) and β-actin (*Actb*). The sequences or commercial references of the primers used are listed in Table S5.

### Statistical analysis

All images shown are representative, and all the data are expressed as the means ± SEMs. The number of tumors included in each experiment is indicated in the corresponding figure legend. All graphical representations and statistical analyses were performed using *Graph Pad 10.4.0* software. qPCR results are represented in box plots that show the median and the 25–75th percentiles, with whiskers showing the 10–90th percentiles. Histology quantification from human samples is represented in violin plots that show the median. Outliers were removed using the “Identify outliers” tool of the software. The Kolmogorov–Smirnov normality test was applied to the datasets before statistical comparisons. Unpaired t-test was used for Gaussian distributed datasets, whereas the Mann–Whitney test was used as a nonparametric test. Angiogenesis/co-option vascularization in lung tumors and hot/cold tumors in human samples were compared using Fisher’s exact test. In all cases, values were considered statistically significant when the p-value < 0.05 and are indicated with asterisks (*p < 0.05; **p < 0.01; ***p < 0.001; ****p < 0.0001).

## Results

### Continuous endoglin overexpression increases hypoxia within LLC subcutaneous tumors

**Fig. 1:**
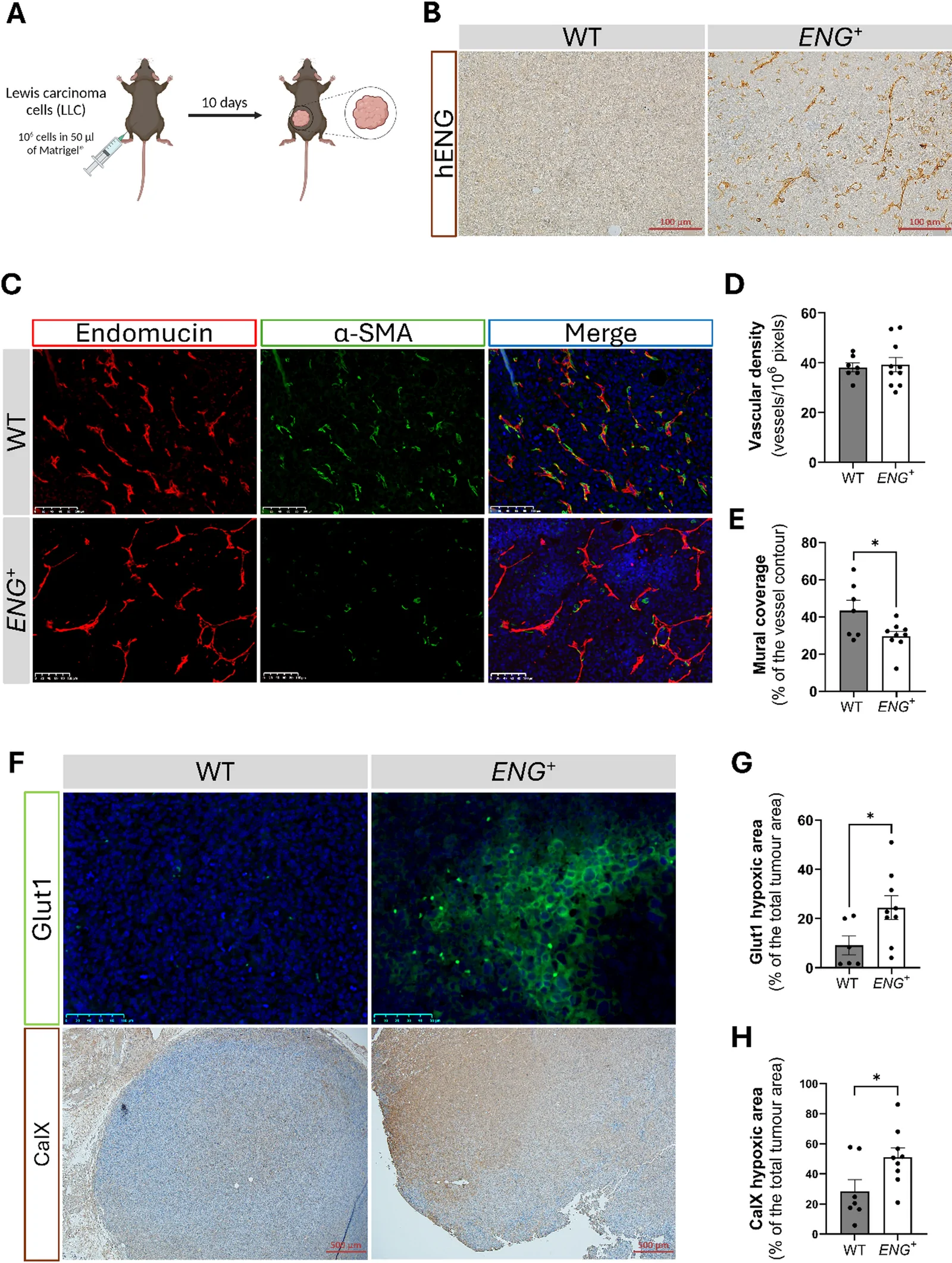
Endoglin overexpression decreases vessel maturation and increases hypoxia in tumors in a murine LLC subcutaneous tumor model. (**A**) Transgenic mice that overexpress endoglin (*ENG*^*+*^) and wild-type C57BL/6J mice (WT), as controls, were used to generate a subcutaneous xenograft tumor model with Lewis lung carcinoma cells (LLC). In this model, 10^6^ cells in 50 µl of Matrigel^®^ were subcutaneously inoculated in the flank of the mice, which were euthanized after 10 days. Created with BioRender.com. (**B**) Human endoglin immunostaining in tumor tissue. (**C**) Immunofluorescence of the vessel marker Endomucin (red) and the mural cell marker α-SMA (green). (**D**) Endoglin levels do not modify vascular density [n(WT) = 7; n(*ENG*^*+*^) = 9; *p* = 0,9419]. (**E**) Tumors from *ENG*^*+*^ mice have more immature vessels than WT mice [n(WT) = 7; n(*ENG*^*+*^) = 9; *p-value < 0,05]. (**F**) Immunofluorescence of the Glut1 marker and immunohistochemistry of the CaIX marker to study the hypoxic areas. (**G**) Tumors from *ENG*^*+*^ mice show a larger hypoxic area for the marker Glut1 than WT mice [n(WT) = 6; n(*ENG*^*+*^) = 9; *p-value < 0,05], (** H**) also for the CaIX marker [n(WT) = 7; n(*ENG*^*+*^) = 9; *p-value < 0,05].

In this work, we first used a xenograft model in which LLC cells with Matrigel^®^ are subcutaneously injected into the flank of *ENG*^*+*^ and WT mice (Fig. 1A). Immunohistochemical analysis revealed that only vessel-like structures, together with some infiltrating cells, are positive for human endoglin in the *ENG*^+^ mice (Fig. 1B). We analyzed vascular density and maturation by double immunofluorescence of the blood vessel marker endomucin and the mural cell marker alpha smooth muscle actin (αSMA)^25^. No differences in the number of endomucin-labeled blood vessels were found (Fig. 1C-D), but the average coverage of these vessels with αSMA-positive cells was significantly lower in tumors developed in *ENG*^*+*^ than in the WT mice (Fig. 1C and E).

It is widely described that the lack of maturation of tumor blood vessels results in a dysfunctional and fenestrated vasculature that facilitates the intravasation of tumor cells, the generation of metastases and does not allow proper irrigation of tumors, leading to hypoxia^26^. To analyze the presence of hypoxic areas, we performed histological analyses of the hypoxic markers glucose transporter 1 (Glut1)^27^ and carbonic anhydrase IX (CaIX)^5^. We observed that the hypoxic area is larger in the subcutaneous tumors developed in *ENG*^*+*^ mice than in those in WT mice (Fig. 1F-H), confirming that incomplete maturation of blood vessels in *ENG*^*+*^ mice may affect the perfusion of the tumors and impair their correct oxygenation.

### Subcutaneous tumors developed in* ENG*^+^ mice show immunosuppressive tumor microenvironment

Tumor hypoxia has been linked to a worse prognosis for several reasons, including that it can affect the recruitment and functionality of different immune cells to the TME. As a preliminary analysis of tumor immune cell infiltration, we analyzed the presence of different immune populations in subcutaneous tumors by flow cytometry of a single-cell suspension from the tumor. Before performing the tumor analysis, we confirmed, via peripheral blood analysis, that *ENG*^*+*^ mice did not show alterations in the analyzed populations (Figure S2).

Following the analysis strategy outlined in Figure S1, we identified the most characteristic leukocyte populations: T lymphocytes (identifying CD4 and CD8), B lymphocytes, natural killer cells (NK), monocytes and neutrophils or MDSCs. Our results reveal a significant reduction in the number of NK cells in the tumors developed in *ENG*^*+*^ mice (Figure S3). Moreover, we also observed a clear trend toward less infiltration of CD8^+^ T cells, B cells and monocytes, but more infiltration of neutrophils or MDSCs (Figure S3).

**Fig. 2:**
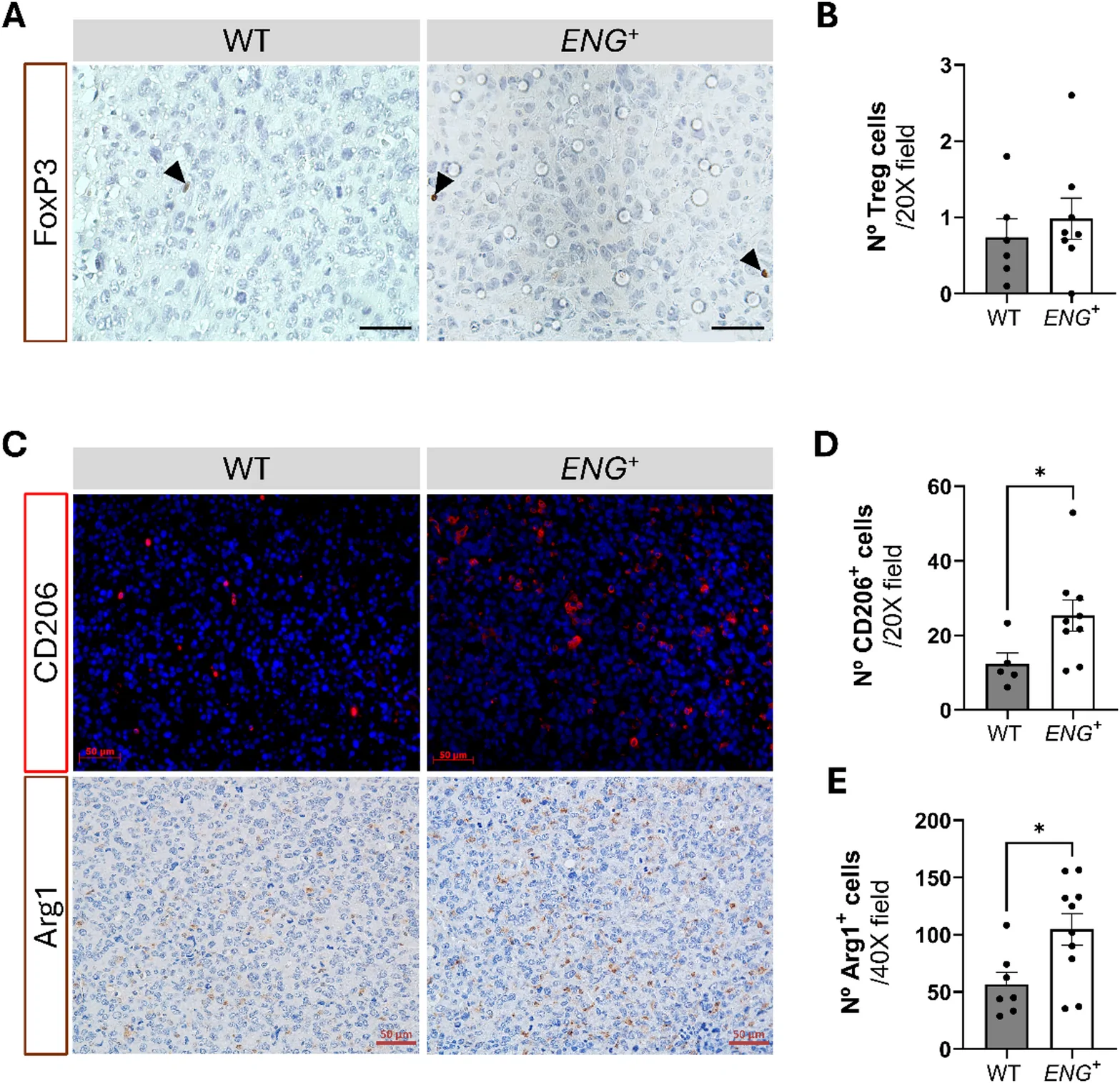
Endoglin overexpression favors protumoral components in the microenvironment of subcutaneous tumors. (**A**) FoxP3 immunostaining showing Treg lymphocytes infiltration. (**B**) Quantification of the number of Treg cells [n(WT) = 6 tumours, n(*ENG*^*+*^) = 8 tumours, *p* = 0,5277]. (**C**) M2 TAM infiltration observed by CD206 immunofluorescence (red) and Arg1 immunostaining. (**D**) Tumours from *ENG*^*+*^ mice show more infiltration of M2 TAM than tumours developed in WT mice, for both CD206 marker [n(WT) = 5, n(*ENG*^*+*^) = 9; *p-value < 0,05], and (**E**) Arg1 marker [n(WT) = 7, n(*ENG*^*+*^) = 10; *p-value < 0,05].

Once we verified that there were changes in the tumor immune populations, we studied the most relevant cell populations in the generation of the tumor immuno-microenvironment via immunohistochemistry. To analyze the presence of Tregs, we performed immunohistological staining of FoxP3, a characteristic marker of this cell type. We found a trend toward a greater number of these immunosuppressive cells in tumors from *ENG*^*+*^ mice than in those from WT mice, but the differences are not statistically significant (Fig. 2A-B). Moreover, to determine the infiltration of protumor M2 TAMs, we analyzed the expression of the specific markers CD206 and Arg1. In line with our hypothesis and the results already described the number of M2 TAMs in the subcutaneous tumors in *ENG*^*+*^ mice is greater than in WT mice (Fig. 2C-E).

**Fig. 3:**
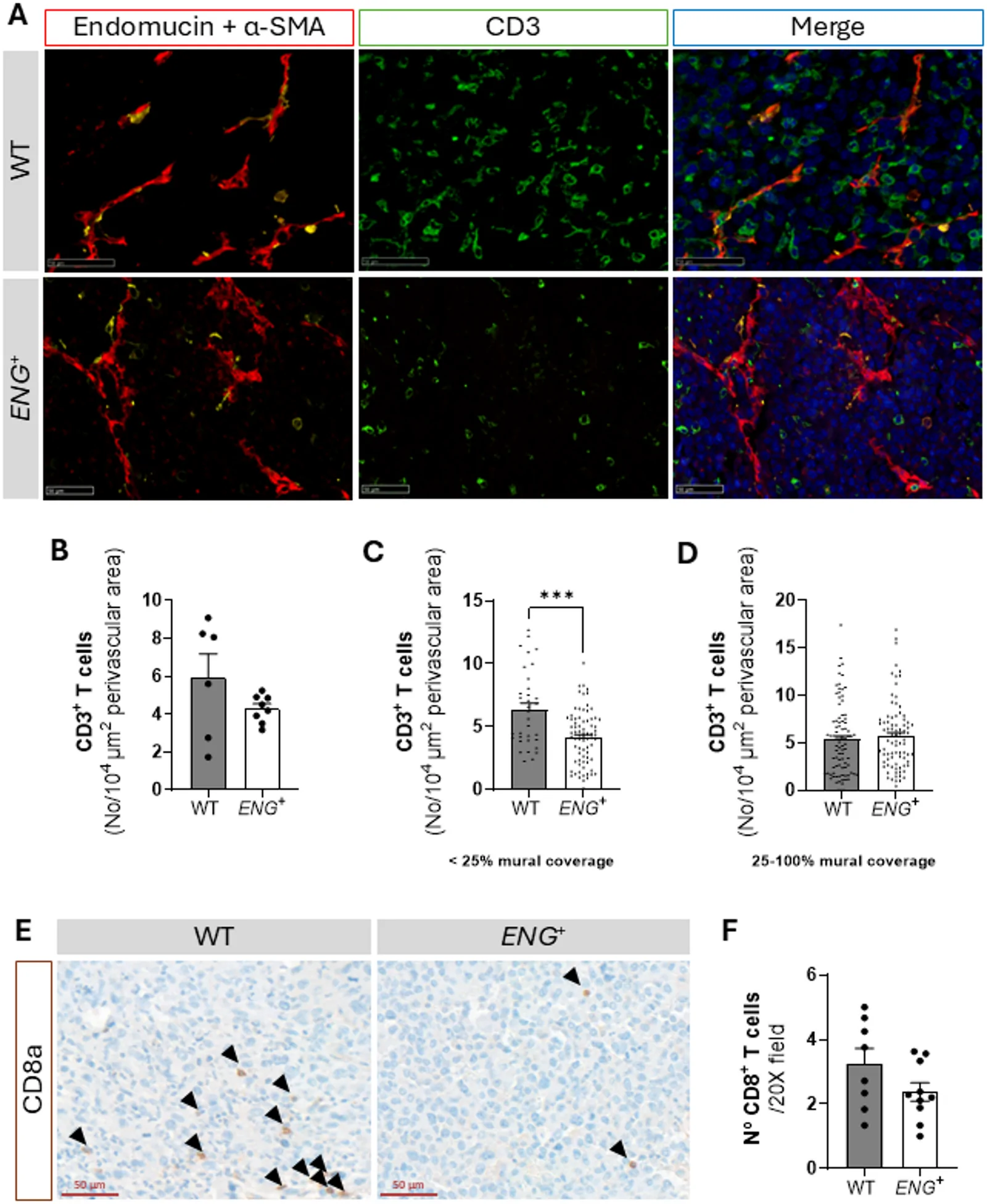
Endoglin overexpression prevents infiltration of antitumoral components in subcutaneous tumors. (**A**) Immunofluorescence of the vessel marker Endomucin (red), mural cell marker α-SMA (yellow) and CD3^+^ lymphocytes (green). (**B**) Quantification of perivascular CD3^+^ T cells [n(WT) = 6 tumors, n(*ENG*^*+*^) = 8 tumors; *p-value < 0,05]. (**C-D**) Quantification of perivascular CD3^+^ T cells [n(WT) = 120 areas (from 6 tumors), n(*ENG*^*+*^) = 160 areas (from 8 tumors); *p-value < 0,05], (**D**) around vessels with less than 25% mural coverage [n(WT) = 34 areas (from 6 tumors), n(*ENG*^*+*^) = 76 areas (from 7 tumors); ****p-value < 0,0001], and (**D**) around vessels with 25–100% mural coverage [n(WT) = 86 areas (from 6 tumors), n(*ENG*^*+*^) = 84 areas (from 7 tumors); *p* = 0,5418]. (**E**) CD8a immunostaining showing cytotoxic lymphocyte infiltration. (**F**) Quantification of the number of CD8^+^ cells from infiltrated areas [n(WT) = 8 tumors, n(*ENG*^*+*^) = 10 tumors, *p < 0,05].

On the other hand, we also studied the presence of cells that inhibit tumor growth. We analyzed the presence of perivascular CD3^+^ lymphocytes. We observed that the tumors developed in *ENG*^*+*^ mice have fewer perivascular lymphocytes (Fig. 3A-B) and that these differences are markedly greater if we looked only at vessels with less than 25% mural coverage (Fig. 3A-D). Although CD3 is a pan-lymphocyte marker expressed by different subpopulations and maturational stages of lymphocytes, these perivascular cells are mainly cytotoxic lymphocytes^28^. To confirm that endoglin overexpression actually reduces the infiltration of these lymphocytes, which we previously observed via flow cytometry, we performed CD8 immunohistochemical staining. As expected, tumors developed in *ENG*^*+*^ mice have a lower number of cytotoxic lymphocytes than those developed in WT mice (Fig. 3E-F).

Finally, we analyzed the expression of some antitumor cytokines by quantitative PCR, such as tumor necrosis factor (*Tnf*), interferon gamma (*Ifng*), interleukin 2 (*Il2*) and IL-12a (*Il12a*), and protumor cytokines, such as IL-1b (*Il1b*), IL-10 (*Il10*), IL-6 (*Il6*) and stromal cell-derived factor 1 (*Cxcl12*), and the expression of cyclooxygenase 2 (*Cox2*) (Figure S4). Taken together, except for *Il12a*, endoglin promotes the expression of protumoral cytokines, including *Il6*, *Cxcl12* and *Cox2* (Figure S4).

### Continuous endoglin overexpression increases hypoxia and shows immunosuppressive tumor microenvironment in lung tumors

**Fig. 4:**
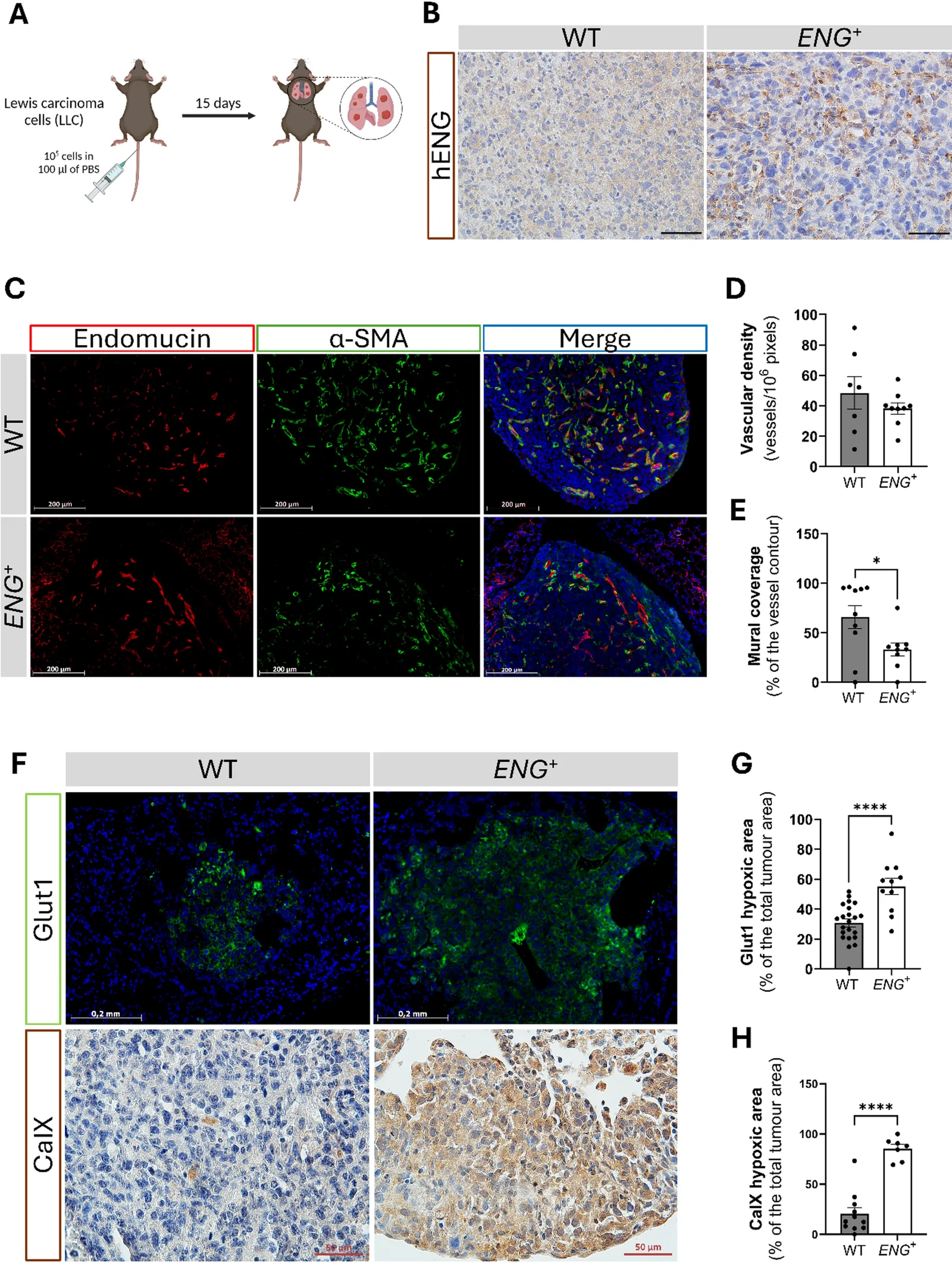
Endoglin overexpression decreases vessel maturation and increases hypoxia in lung tumors. (**A**) Transgenic mice that overexpress endoglin (*ENG*^*+*^) and wild-type C57BL/6J mice (WT), as controls, were used to generate a murine lung tumor model with Lewis lung carcinoma cells (LLC). In this model, 10^5^ cells resuspended in 100 µl of PBS were injected intravenously to travel through the bloodstream and implant in the lungs, where they generate the tumor. These mice were euthanized after 15 days. Created with BioRender.com. (**B**) Human endoglin immunostaining in the tumor tissue [scale bar: 50 μm]. (**C**) Immunofluorescence of the vessel marker Endomucin (red) and mural cell marker α-SMA (green). (**D**) Endoglin levels do not modify vascular density [n(WT) = 7; n(*ENG*^*+*^) = 10; *p* = 0,8868]. (**E**) Tumors from *ENG*^*+*^ mice have more immature vessels than those from WT mice [n(WT) = 10; n(*ENG*^*+*^) = 9; *p-value < 0,05]. (**F**) Immunofluorescence of the Glut1 marker and immunohistochemistry of the CaIX marker to study the hypoxic areas. (**G**) Tumors from *ENG*^*+*^ mice have a larger hypoxic area than those from WT mice [n(WT) = 22; n(*ENG*^*+*^) = 11; ***p-value < 0,0001], (**H**) also for the CaIX marker [n(WT) = 11 tumors; n(*ENG*^*+*^) = 7 tumors; ****p-value < 0,0001].

To test the effect of the overexpression of endoglin in tumors within the lung microenvironment, LLC cells were intravenously injected so that they travel through the bloodstream and implant in the lungs, where they generate tumors^24^(Fig. 4A). The lung is a highly vascularized tissue, where tumors are able to use at least two forms of vascularisation: vascular co-option, in which the tumor cells proliferate around existing blood vessels in the host tissue^29^, and angiogenesis. To differentiate between lung tumors with angiogenic vasculature and those with vascular co-option, double immunofluorescence of endomucin (blood vessel marker) and podoplanin (alveolar type I cell marker) was used. Co-opted areas, characterized by the maintenance of normal lung parenchyma with a “honeycomb” morphology^30^, present both markers inside the tumor mass; whereas angiogenic areas are characterized by the destruction of normal lung parenchyma and disorganized neovascularization within the tumor. Our results demonstrate that angiogenic, co-option and mixed tumors, which share both vascularization patterns, can be found in this model (Figure S5). Given that WT and *ENG*^*+*^ mice equally develop both angiogenic and co-option vascular profiles (Figure S5), in accordance with our objective, we focused only on angiogenic tumors. In this work, we considered an angiogenic tumor when the angiogenic area occupied at least 35% of the total tumor area.

These angiogenic vessels present a similar structure to those observed in the subcutaneous xenograft model, so we first analyzed vascular density and vessel maturation by endomucin and α-SMA double immunofluorescence. As in the subcutaneous model, lung tumors developed in *ENG*^*+*^ and WT mice have a similar density of vasculature, with vessels from *ENG*^*+*^ mice having less mural coverage than those from WT mice (Fig. 4C-E). Accordingly, when we analyzed hypoxia, we detected that hypoxic areas are larger in *ENG*^*+*^ lung tumors than those developed in WT mice (Fig. 4F-H), confirming that endoglin overexpression also impairs correct oxygenation in this tumor model.

**Fig. 5:**
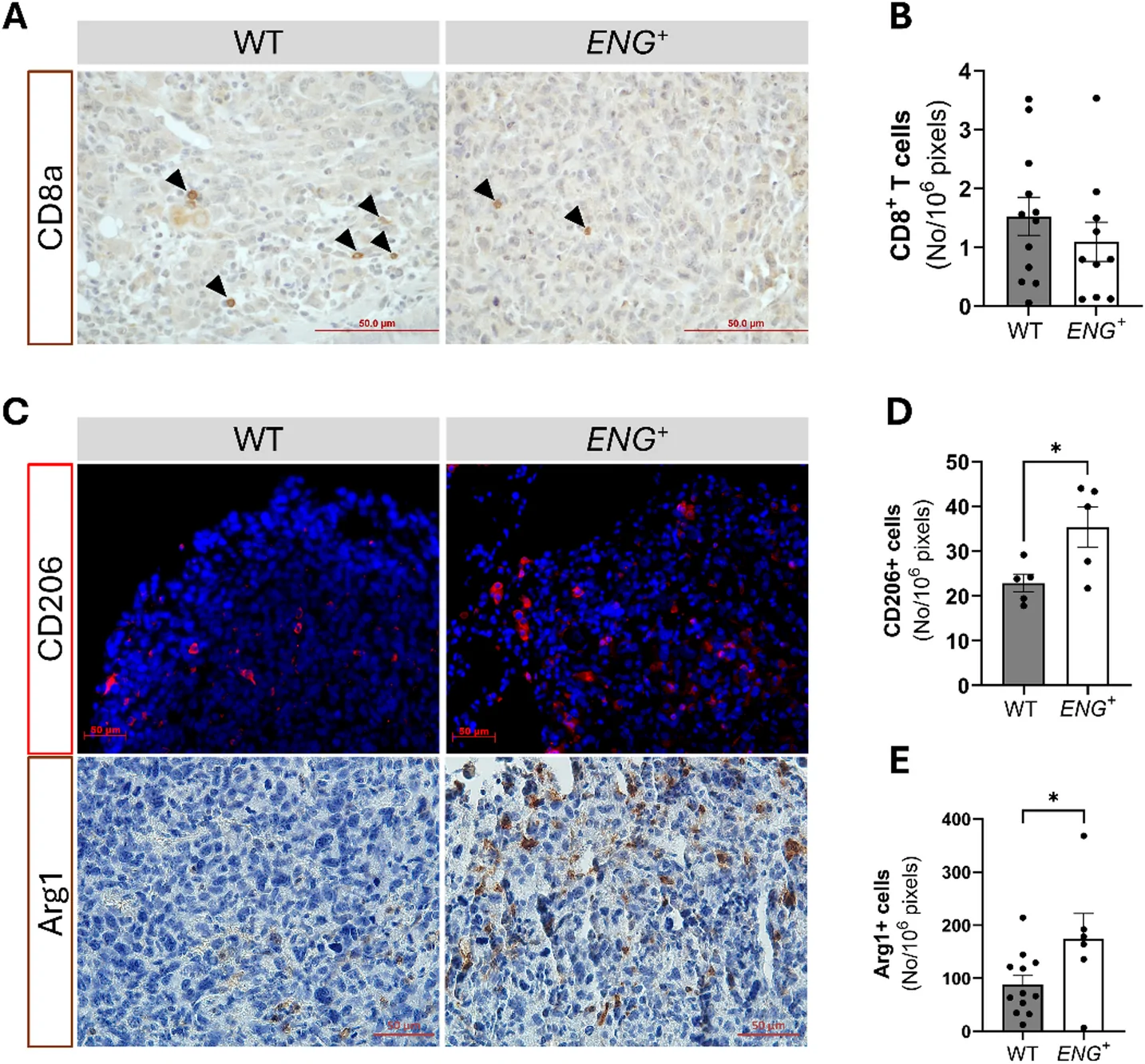
Endoglin overexpression modifies the infiltration of leukocyte populations in angiogenic lung tumors. (**A**) CD8α immunostaining showing CD8^+^ lymphocyte infiltration. (**B**) Quantification of the number of CD8^+^ cells from infiltrated areas [n(WT) = 13, n(*ENG*^*+*^) = 10, *p* = 0,4176]. (**C**) CD206 immunofluorescence showing M2 TAM infiltration (red). (**D**) Tumors from *ENG*^*+*^ mice show greater infiltration of M2 TAMs than did those from WT mice [n(*WT*) = 5, n(*ENG*^*+*^) = 5; *p-value < 0,05], and also for (**E**) Arg1 marker [n(*WT*) = 12, n(*ENG*^*+*^) = 6; *p < 0,05].

We also analyzed the TME composition of these tumors via histological studies. Although lymphocyte infiltration is virtually undetectable, due to the short duration of tumor development and the lung tumor model used^31^, a trend toward less infiltration of antitumor CD8^+^ lymphocytes is observed in endoglin-overexpressing lung tumors (Fig. 5A-B). Conversely, and validating the results observed in the subcutaneous model, histological analyses of CD206 and Arg1 revealed that lung tumors that developed in *ENG*^*+*^ mice have a higher number of pro tumor M2 TAMs than WT tumors (Fig. 5C-E).

### High endoglin expression is associated with an immunosuppressive TME in solid angiogenic samples of human lung adenocarcinoma (LUAD)

To analyze the clinical significance of endoglin expression in determining the TME, we study TME in lung adenocarcinomas (LUAD) previously classified as solid (according to the WHO 2021 classification of lung adenocarcinomas) and as angiogenic. Similar to what was done in mice, we used CD8, FoxP3 and CD206 as markers of the type of TME.

**Fig. 6:**
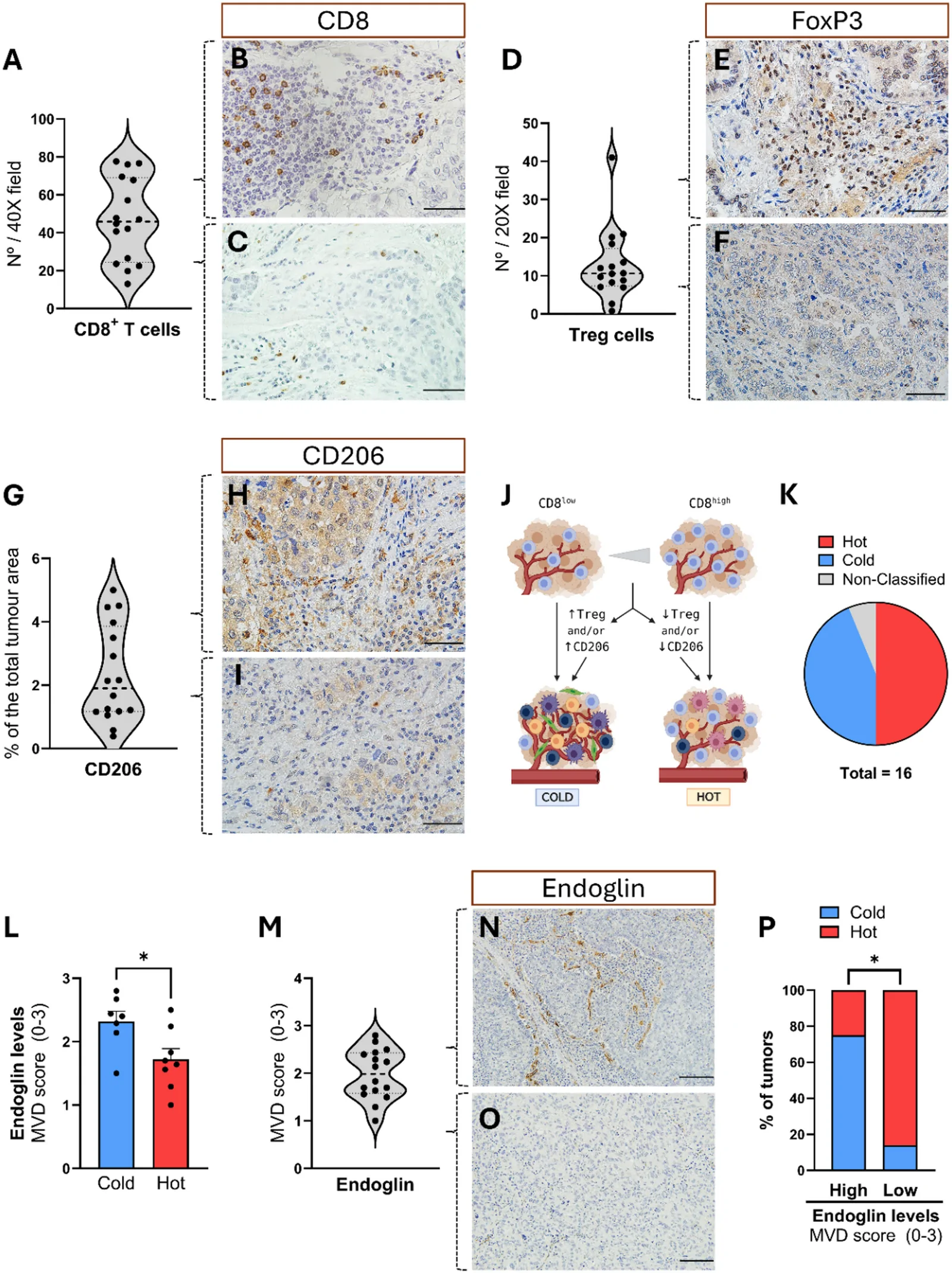
Characterization of the tumor microenvironment in solid angiogenic samples of human lung adenocarcinoma. Characterization of the tumor microenvironment in solid angiogenic samples of human lung adenocarcinoma. (**A**) Distribution of the samples according to the number of CD8^+^ T cells per field. (**B**) CD8 immunostaining showing high and (**C**) low infiltration of CD8^+^ T cells [scale bar 20 μm]. (**D**) Distribution of the samples according to the number of Treg cells per field. (**E**) FoxP3 immunostaining showing high and (**F**) low infiltration of Treg cells [scale bar 50 μm]. (**G**) Distribution of the samples according to the area positive for CD206 with respect to the total tumor area. (**H**) CD206 immunostaining showing high and (**I**) low infiltration of M2 TAMs [scale bar 50 μm]. (**J**) Algorithm used for characterizing the type of TME: cold when CD8^+^ T-cell infiltration is low and hot when it is high, but for medium CD8^+^ T-cell, infiltration of Treg and M2 TAMs was also evaluated. Created with BioRender.com. (**K**) Classification of samples according to their type of TME (cold/hot/non-classified). (**L**) Tumors identified as cold correlate with higher levels of endoglin [n(cold) = 7, n(hot) = 8; *p < 0,05]. (**M**) Distribution of the different samples according to their MVD score. (**N**) CD105 immunostaining showing high and (**O**) low endoglin levels [scale bar 50 μm]. (**P**) Tumors with high endoglin levels are identified as cold, whereas tumors with low endoglin levels are hot according to their composition of the TME [n(cold) = 7, n(hot) = 8; *p < 0,05].

The study of CD8^+^ cell infiltration shows that human tumors clustered into 3 subgroups, as shown in the violin plot (Fig. 6A). Some tumors had high infiltration (Fig. 6B), while others had very little infiltration (Fig. 6C), and a subgroup had intermediate infiltration. We therefore used the median to determine whether the values were high or low since, as explained above, high CD8^+^ lymphocyte infiltration is associated with a hot TME, whereas low infiltration is associated with a cold TME. To discriminate tumors with intermediate levels, we analyzed the infiltration of FoxP3- and CD206-positive cells associated with cold tumors. Similarly, we classified the samples as high or low, using the median in the violin plots (Fig. 6D and G), as we found tumors with high and low expression of these markers (Fig. 6E-F and H-I).

Using the algorithm explained in the methods (Fig. 6J), we were able to classify 8 tumors as “hot” and 7 as “cold”. In one of the tumors, the result indicated by FoxP3 and CD206 was opposite to that of CD8, so we termed that tumor “unclassified” (Fig. 6K).

We analyzed endoglin expression and assigned a score according to staining and MVD, and our results show that endoglin levels are higher in tumors classified as cold than in those classified as hot (Fig. 6L). On the other hand, when the endoglin score was represented in a violin plot, two highly defined populations were detected, allowing the tumors to be classified as Eng^high^ or Eng^low^ (Fig. 6M-O). Interestingly, lung adenocarcinomas classified as Eng^high^ correlate significantly more with those classified as cold, whereas Eng^low^ tumors correlate significantly more with those classified as hot (Fig. 6P).

## Discussion

The recent, albeit unofficial, classification of tumors into two categories, “hot”, referred to as inflamed, and “cold”, referred to as immune-desert, is becoming increasingly widespread. This categorization depends mainly on the characteristics of its TME^32,33^: hot tumors are characterized by high infiltration of CD8^+^ T cells, as opposed to cold tumors, which are characterized by lower infiltration of CD8^+^ T cells and the presence of immunosuppressive cells such as MDSCs and Tregs. This composition of the TME affects tumor prognosis and response to immunotherapy, as cold tumors benefit less from this therapeutic approach and have a worse prognosis than hot tumors do, which usually respond well^32–34^. Cold tumors are also associated with poor vascular function, leading to poor perfusion and favoring the development of hypoxia, which promotes the survival of the most aggressive cells and the recruitment of anti-inflammatory cells^4,12,15–17^.

In this study, we used two murine tumor models with LLC cells: a xenograft model implanted subcutaneously and a lung model after intravenous injection. Tumors created from LLC cells have been shown to have an immunosuppressive TME^35,36^. Our results show that endoglin overexpression increases this phenotype even more, as tumors developed in *ENG*^*+*^ mice present fewer anti-tumor cells, such as CD8^+^ lymphocytes and NK cells, and more pro-tumor Treg lymphocytes and M2 TAMs. The immunosuppressive phenotype is also reflected in TME cytokines, with increased expression of *Cxcl12*, *Il-6* and *Cox2*, which promote tumor cell proliferation, migration, invasion and recruitment of M2 TAMs and Tregs^37–39^. The existence of an immunosuppressive TME in tumors with endoglin overexpression is consistent with the increased hypoxia observed in these tumors.

This correlation has been highlighted when we have studied the TME and endoglin expression in human LUAD samples. As explained above, CD8^+^ cell infiltration is the main feature that allows tumors to be categorized as hot or cold^12,32–34^. For borderline phenotypes, we also used the presence of immunosuppressive cells, Tregs or M2 TAMs, as confirmation of the tumor phenotype^12,32–34^. Subsequent analysis of microvascular endoglin levels revealed that tumors classified as cold had higher endoglin levels than hot tumors. In addition, we observed that the samples were grouped as Eng^high^ or Eng^low^ and that Eng^high^ corresponded to cold tumors, whereas Eng^low^ corresponded to hot tumors.

**Fig. 7:**
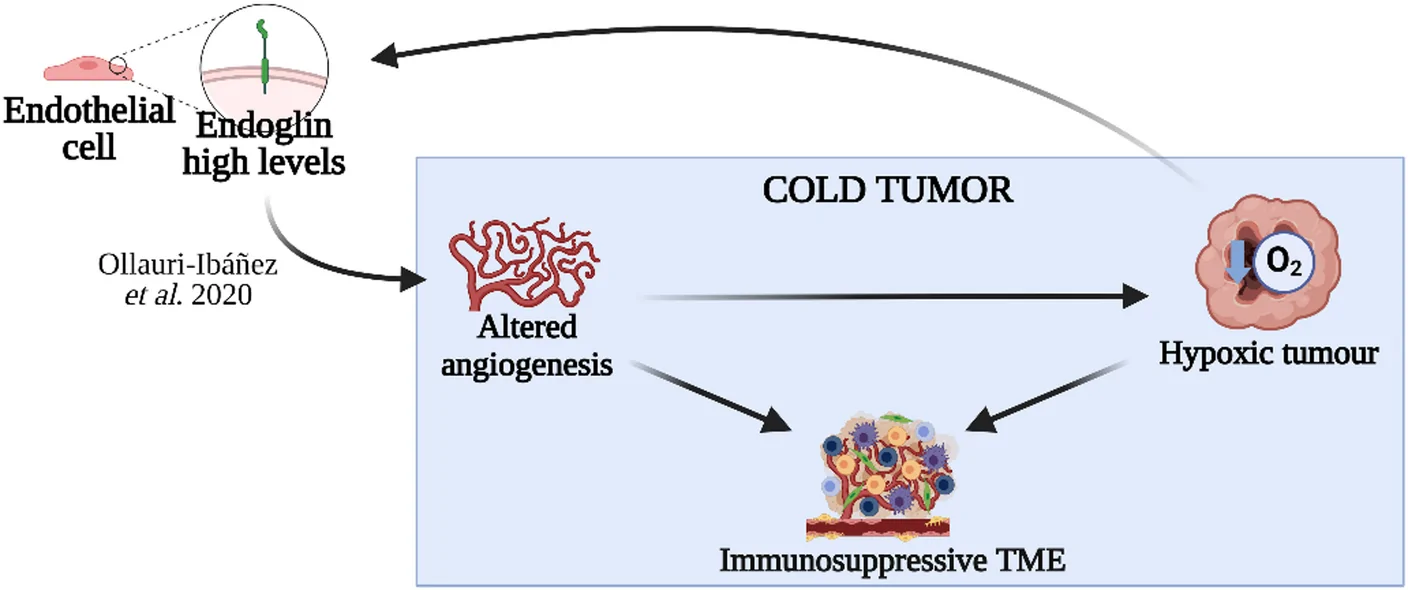
Increased endothelial endoglin levels are related to altered angiogenesis, which results in hypoxia, that is associated with an immunosuppressive TME. Hypoxia also induces endoglin expression, which may also contribute to the sustained elevated endoglin levels.

Several authors have shown that high microvessel endoglin levels are associated with a poor prognosis in cancers such as NSCLC^40^, hepatocellular carcinoma^41^, astrocytic tumors^42^ and breast carcinoma^43^, among others. Traditionally, it was assumed that the poorer prognosis of these tumors was due to increased angiogenesis leading to increased tumor growth. However, in a previous study, we demonstrated that endoglin overexpression does not increase angiogenesis but impedes proper capillary maturation. This impaired angiogenesis results in more permeable vessels that favor intravasation of tumor cells, generating metastases^22^. In this work, we demonstrate that tumors with elevated levels of endoglin are also associated with a hypoxic and immunosuppressive microenvironment, allowing us to classify these tumors as cold. The abundant literature on this subject allows us to propose that the alterations in angiogenesis caused by elevated endoglin expression^22^ are related to the appearance of hypoxic areas. Both alterations, especially hypoxia, prevent the recruitment of anti-tumor cells and favor the recruitment of pro-tumor cells, which defines the immunosuppressive microenvironment characteristic of cold tumors. In addition, hypoxia is able to induce endoglin expression, which could lead to a positive feedback loop^44^(Fig. 7). One limitation of this study is the use of a ubiquitous endoglin overexpression model rather than an endothelial-specific transgene, which prevents exclusive attribution of the effects to endothelial endoglin. Nevertheless, our prior *in vitro and in vivo* data show that endothelial cells overexpressing endoglin remain in an activated phenotype that impairs mural cell attachment^22^, the diminished CD3^+^ cell infiltration in the perivascular area of the immature vessel observed in this study, and the numerous clinical studies that localize endoglin predominantly to the tumor vasculature and associate higher vascular endoglin with poorer prognosis^20,21^ supports a primarily vascular contribution, while not excluding additional stromal inputs.

In conclusion, our work proposes that endoglin could be used for both the diagnosis and treatment of cancer. In diagnosis, the use of endoglin as a biomarker could help to determine whether a tumor is cold or hot, making it possible to rule out cases in which immunotherapy would not be effective. In therapy, the use of anti-endoglin antibodies, such as carotuximab (TRC105), could help reduce endothelium activation, generating better blood vessels that could improve the delivery of other drugs to the tumor, decrease hypoxia, promote an immunostimulatory TME and reduce metastasis generation, resulting in a better response to immunotherapy and an overall better prognosis.

## Supplementary Information

Below is the link to the electronic supplementary material.

Supplementary Material 1

## Data Availability

The datasets used and/or analyzed during the current study are available from the corresponding author upon reasonable request.

## References

[1] Folkman, J., Merler, E., Abernathy, C. & Williams, G. Isolation of a tumor factor responsible for angiogenesis. *J. Exp. Med.* **133**, 275–288. 10.1084/jem.133.2.275 (1971). 4332371 10.1084/jem.133.2.275 PMC2138906

[2] Huang, H. et al. Development and validation of a nomogram for evaluating the prognosis of immunotherapy plus antiangiogenic therapy in non-small cell lung cancer. *Cancer Cell. Int.* **22**, 261. 10.1186/s12935-022-02675-y (2022). 35989349 10.1186/s12935-022-02675-y PMC9394085

[3] Remon, J. et al. ANtiangiogenic Second-line Lung cancer Meta-Analysis on individual patient data in non-small cell lung cancer: ANSELMA. *Eur. J. Cancer*. **166**, 112–125. 10.1016/j.ejca.2022.02.002 (2022). 35286903 10.1016/j.ejca.2022.02.002

[4] Graeber, T. G. et al. Hypoxia-mediated selection of cells with diminished apoptotic potential in solid tumours. *Nature* **379**, 88–91. 10.1038/379088a0 (1996). 8538748 10.1038/379088a0

[5] Ilie, M. et al. High levels of carbonic anhydrase IX in tumour tissue and plasma are biomarkers of poor prognostic in patients with non-small cell lung cancer. *Br. J. Cancer*. **102**, 1627–1635. 10.1038/sj.bjc.6605690 (2010). 20461082 10.1038/sj.bjc.6605690 PMC2883156

[6] Wolchok, J. Putting the Immunologic Brakes on Cancer. *Cell* **175**, 1452–1454. 10.1016/j.cell.2018.11.006 (2018). 30500529 10.1016/j.cell.2018.11.006

[7] Ribas, A. & Wolchok, J. D. Cancer immunotherapy using checkpoint blockade. *Science* **359**, 1350–1355. 10.1126/science.aar4060 (2018). 29567705 10.1126/science.aar4060 PMC7391259

[8] Paz-Ares, L. G. et al. First-Line Nivolumab Plus Ipilimumab in Advanced NSCLC: 4-Year Outcomes From the Randomized, Open-Label, Phase 3 CheckMate 227 Part 1 Trial. *J. Thorac. Oncol.* **17**, 289–308. 10.1016/j.jtho.2021.09.010 (2022). 34648948 10.1016/j.jtho.2021.09.010

[9] Schadendorf, D. et al. STARBOARD: encorafenib + binimetinib + pembrolizumab for first-line metastatic/unresectable BRAF V600-mutant melanoma. *Future Oncol.* **18**, 2041–2051. 10.2217/fon-2021-1486 (2022). 35272485 10.2217/fon-2021-1486

[10] Brown, L. J. et al. Site-specific response and resistance patterns in patients with advanced non-small-cell lung cancer treated with first-line systemic therapy. *Cancers (Basel)*. 10.3390/cancers16112136 (2024). 38893255 10.3390/cancers16112136 PMC11172392

[11] Xu, Z. et al. Real–world evidence of advanced non–small cell lung carcinoma treated with an immune checkpoint inhibitor plus chemotherapy. *Oncol. Lett.* **28**, 405. 10.3892/ol.2024.14538 (2024). 38983127 10.3892/ol.2024.14538 PMC11228919

[12] De Guillebon, E. et al. Beyond the concept of cold and hot tumors for the development of novel predictive biomarkers and the rational design of immunotherapy combination. *Int. J. Cancer*. **147**, 1509–1518. 10.1002/ijc.32889 (2020). 31997345 10.1002/ijc.32889

[13] Anderson, N. M. & Simon, M. C. Tumor Microenvironment. *Curr. Biol.* **30**, R921–R925. 10.1016/j.cub.2020.06.081 (2020). 32810447 10.1016/j.cub.2020.06.081 PMC8194051

[14] Ollauri-Ibáñez, C., Ayuso-Íñigo, B. & Pericacho, M. Hot and cold tumors: is endoglin (CD105) a potential target for vessel normalization? *Cancers (Basel)*. **13**, 1552. 10.3390/cancers13071552 (2021). 33800564 10.3390/cancers13071552 PMC8038031

[15] Colombani, T. et al. Hypoxia-inducing cryogels uncover key cancer-immune cell interactions in an oxygen-deficient tumor microenvironment. *Bioact Mater.* **29**, 279–295. 10.1016/j.bioactmat.2023.06.021 (2023). 37600932 10.1016/j.bioactmat.2023.06.021 PMC10432785

[16] Fan, P. et al. Alleviating hypoxia to improve cancer immunotherapy. *Oncogene* **42**, 3591–3604. 10.1038/s41388-023-02869-2 (2023). 37884747 10.1038/s41388-023-02869-2

[17] Gide, T. N. et al. da, Distinct immune cell populations define response to anti-PD-1 monotherapy and anti-PD-1/Anti-CTLA-4 combined therapy. Cancer Cell. 35, 238–255 10.1016/j.ccell.2019.01.003 (2019). 30753825 10.1016/j.ccell.2019.01.003

[18] ten Dijke, P., Goumans, M. J. & Pardali, E. Endoglin in angiogenesis and vascular diseases. *Angiogenesis* **11**, 79–89. 10.1007/s10456-008-9101-9 (2008). 18283546 10.1007/s10456-008-9101-9

[19] Rossi, E. & Bernabeu, C. Novel vascular roles of human endoglin in pathophysiology. *J. Thromb. Haemost.* **21**, 2327–2338. 10.1016/j.jtha.2023.06.007 (2023). 37315795 10.1016/j.jtha.2023.06.007

[20] Nassiri, F. et al. Endoglin (CD105): a review of its role in angiogenesis and tumor diagnosis, progression and therapy. *Anticancer Res.* **31**, 2283–2290 (2011). 21737653

[21] Paauwe, M., ten Dijke, P. & Hawinkels, L. J. A. C. Endoglin for tumor imaging and targeted cancer therapy. *Expert Opin. Ther. Targets*. **17**, 421–435. 10.1517/14728222.2013.758716 (2013). 23327677 10.1517/14728222.2013.758716

[22] Ollauri-Ibáñez, C. et al. Continuous endoglin (CD105) overexpression disrupts angiogenesis and facilitates tumor cell metastasis. *Angiogenesis* **23**, 231–247. 10.1007/s10456-019-09703-y (2020). 31897911 10.1007/s10456-019-09703-y PMC7160077

[23] Oujo, B. et al. L-Endoglin overexpression increases renal fibrosis after unilateral ureteral obstruction. *PLoS One*. **9**, e110365. 10.1371/journal.pone.0110365 (2014). 25313562 10.1371/journal.pone.0110365 PMC4196986

[24] Janker, F., Weder, W., Jang, J. H. & Jungraithmayr, W. Preclinical, non-genetic models of lung adenocarcinoma: a comparative survey. *Oncotarget* **9**, 30527–30538. 10.18632/oncotarget.25668 (2018). 30093966 10.18632/oncotarget.25668 PMC6078138

[25] Armulik, A., Genové, G. & Betsholtz, C. Pericytes: developmental, physiological, and pathological perspectives, problems, and promises. *Dev. Cell.* **21**, 193–215. 10.1016/j.devcel.2011.07.001 (2011). 21839917 10.1016/j.devcel.2011.07.001

[26] Wong, P. P., Bodrug, N. & Hodivala-Dilke, K. M. Exploring Novel Methods for Modulating Tumor Blood Vessels in Cancer Treatment. *Curr. Biol.* **26**, R1161–R1166. 10.1016/j.cub.2016.09.043 (2016). 27825457 10.1016/j.cub.2016.09.043

[27] Wong, P. P. et al. Dual-action combination therapy enhances angiogenesis while reducing tumor growth and spread. *Cancer Cell.* **27**, 123–137. 10.1016/j.ccell.2014.10.015 (2015). 25584895 10.1016/j.ccell.2014.10.015

[28] Di Tacchio, M. et al. Tumor Vessel Normalization, Immunostimulatory Reprogramming, and Improved Survival in Glioblastoma with Combined Inhibition of PD-1, Angiopoietin-2, and VEGF. *Cancer Immunol. Res.* **7**, 1910–1927. 10.1158/2326-6066.CIR-18-0865 (2019). 31597643 10.1158/2326-6066.CIR-18-0865

[29] Kuczynski, E. A., Vermeulen, P. B., Pezzella, F., Kerbel, R. S. & Reynolds, A. R. Vessel co-option in cancer. *Nat. Rev. Clin. Oncol.* **16**, 469–493. 10.1038/s41571-019-0181-9 (2019). 30816337 10.1038/s41571-019-0181-9

[30] Bridgeman, V. L. et al. Vessel co-option is common in human lung metastases and mediates resistance to anti-angiogenic therapy in preclinical lung metastasis models. *J. Pathol.* **241**, 362–374. 10.1002/path.4845 (2017). 27859259 10.1002/path.4845 PMC5248628

[31] Boumelha, J. et al. An immunogenic model of KRAS-mutant lung cancer enables evaluation of targeted therapy and immunotherapy combinations. *Cancer Res.* **82**, 3435–3448. 10.1158/0008-5472.CAN-22-0325 (2022). 35930804 10.1158/0008-5472.CAN-22-0325 PMC7613674

[32] Galon, J. & Bruni, D. Approaches to treat immune hot, altered and cold tumours with combination immunotherapies. *Nat. Rev. Drug Discov*. **18**, 197–218. 10.1038/s41573-018-0007-y (2019). 30610226 10.1038/s41573-018-0007-y

[33] Raskov, H., Orhan, A., Christensen, J. P. & Gögenur, I. Cytotoxic CD8 + T cells in cancer and cancer immunotherapy. *Br. J. Cancer*. **124**, 359–367. 10.1038/s41416-020-01048-4 (2021). 32929195 10.1038/s41416-020-01048-4 PMC7853123

[34] Trujillo, J. A., Sweis, R. F., Bao, R. & Luke, J. J. T Cell–Inflamed versus Non-T Cell–Inflamed Tumors: A Conceptual Framework for Cancer Immunotherapy Drug Development and Combination Therapy Selection. *Cancer Immunol. Res.* **6**, 990–1000. 10.1158/2326-6066.CIR-18-0277 (2018). 30181337 10.1158/2326-6066.CIR-18-0277 PMC6145135

[35] Mosely, S. I. S. et al. Rational Selection of Syngeneic Preclinical Tumor Models for Immunotherapeutic Drug Discovery. *Cancer Immunol. Res.* **5**, 29–41. 10.1158/2326-6066.CIR-16-0114 (2017). 27923825 10.1158/2326-6066.CIR-16-0114

[36] Mugarza, E. et al. Therapeutic KRASG12C inhibition drives effective interferon-mediated antitumor immunity in immunogenic lung cancers. *Sci. Adv.* **8**, eabm8780. 10.1126/sciadv.abm8780 (2022). 35857848 10.1126/sciadv.abm8780 PMC9299537

[37] Ciummo, S. L. et al. The C-X-C Motif Chemokine Ligand 1 Sustains Breast Cancer Stem Cell Self-Renewal and Promotes Tumor Progression and Immune Escape Programs. *Front. Cell. Dev. Biol.* **9**, 689286. 10.3389/fcell.2021.689286 (2021). 34195201 10.3389/fcell.2021.689286 PMC8237942

[38] Shen, L. et al. Local Blockade of Interleukin 10 and C-X-C Motif Chemokine Ligand 12 with Nano-Delivery Promotes Antitumor Response in Murine Cancers. *ACS Nano*. **12**, 9830–9841. 10.1021/acsnano.8b00967 (2018). 30253648 10.1021/acsnano.8b00967

[39] Wang, S. et al. XIAOPI Formula Inhibits Breast Cancer Stem Cells via Suppressing Tumor-Associated Macrophages/C-X-C Motif Chemokine Ligand 1 Pathway. *Front. Pharmacol.* **10**, 1371. 10.3389/fphar.2019.01371 (2019). 31803057 10.3389/fphar.2019.01371 PMC6874098

[40] Tanaka, F. et al. Evaluation of angiogenesis in non-small cell lung cancer: comparison between anti-CD34 antibody and anti-CD105 antibody. *Clin. Cancer Res.* **7**, 3410–3415 (2001). 11705856

[41] Yao, Y. et al. Endoglin (CD105) expression in angiogenesis of primary hepatocellular carcinomas: analysis using tissue microarrays and comparisons with CD34 and VEGF. *Ann. Clin. Lab. Sci.* **37**, 39–48 (2007). 17311868

[42] Yao, Y., Kubota, T., Takeuchi, H. & Sato, K. Prognostic significance of microvessel density determined by an anti-CD105/endoglin monoclonal antibody in astrocytic tumors: comparison with an anti-CD31 monoclonal antibody. *Neuropathology* **25**, 201–206. 10.1111/j.1440-1789.2005.00632.x (2005). 16193836 10.1111/j.1440-1789.2005.00632.x

[43] Kumar, S. et al. Breast carcinoma: vascular density determined using CD105 antibody correlates with tumor prognosis. *Cancer Res.* **59**, 856–861 (1999). 10029075

[44] Sánchez-Elsner, T., Botella, L. M., Velasco, B., Langa, C. & Bernabéu, C. Endoglin expression is regulated by transcriptional cooperation between the hypoxia and transforming growth factor-beta pathways. *J. Biol. Chem.* **277**, 43799–43808. 10.1074/jbc.M207160200 (2002). 12228247 10.1074/jbc.M207160200

