# Supplementary material for: Endoglin (CD105) overexpression is associated with an immunosuppressive tumor microenvironment in murine lung cancer models and human lung adenocarcinoma
Source: Sci Rep. 2026 Jun 12;16:26896. doi: 10.1038/s41598-026-55711-6 (PMC13518793; doi:10.1038/s41598-026-55711-6)
Supplement: Supplementary file 1 [file 41598_2026_55711_MOESM1_ESM.docx]

**SUPPLEMENTARY MATERIALS**

**Supplementary Table 1:** Cocktail of antibodies for flow cytometry

| **Antibody** | **Dilution** | **Reference** |
| --- | --- | --- |
| Anti-CD3e-BUV615 | 1:40 | BD Biosciences 560804 |
| Anti-CD4-APCH7 | 1:40 | BD Biosciences 560246 |
| Anti-CD8 alpha-PO | 1:50 | Invitrogen MCD0830TR |
| Anti-CD11b-PeCy7 | 1:50 | BD Biosciences 561098 |
| Anti-CD19-PERCPCy5.5 | 1:40 | BD Biosciences 561098 |
| Anti-CD45-PE | 1:40 | BD Biosciences 553081 |
| Anti-Gr1-APC | 1:50 | BD Biosciences 561083 |
| Anti-NK1.1-FITC | 1:100 | BD Biosciences 561082 |

**Supplementary Table 2:** Combination of markers used in flow cytometry to identify each cell type

| **LYMPHOID ORIGIN** | | | |
| --- | --- | --- | --- |
| **T lymphocytes** | CD45^hi^; CD3^+^; NK1.1^-^; CD19^-^; CD11b^-^; FSC^lo^; SSC^lo^ | | |
|  | **Cytotoxic**  CD8^+^; CD4^-^ | **Helper**  CD8^-^; CD4^+^ | **Other maturative stages**  CD8^+^; CD4^+^/CD8^-^; CD4^-^ |
| **B lymphocytes** | CD45^+^; CD19^+^; CD3^-^; NK1.1^-^; Gr1^-^; CD11b^-^; FSC^lo^; SSC^lo^ | | |
| **Natural killers (NKs)** | CD45^+^; NK1,1^+^; CD3^-^; CD19^-^; CD4^-^; CD11b^-^; Gr1^het^; FSC^lo^; SSC^lo^ | | |

| **MYELOID ORIGIN** | |
| --- | --- |
| **Monocytes** | CD45^hi^; CD11b^+^; Gr1^het^; CD19^-^; NK1.1^+^; FSC-SSC🡫 |
| **Neutrophils** | CD45^hi^; CD11b^hi^; Gr1^hi^; CD19^-^; NK1.1^-^; FSC-SSC🡩 |
| **Myeloid-derived suppressor cells (MDSCs)** | CD45^hi^; CD11b^hi^; Gr1^hi^; CD19^-^; NK1.1^-^; FSC-SSC🡫 |

**Supplementary Table 3:** Antibodies used for immunohistochemistry

| **Antibody** | **Dilution** | **Reference** |
| --- | --- | --- |
| Anti-Arg1 | 1:100 | Cell Signaling 93668 |
| Anti-CaIX | 1:50 | Novus NB100-417 |
| Anti-CD8α | 1:100 | Thermo Fisher Scientific 14-0808-80 |
| Anti-Foxp3 | 1:50 | Cell Signaling 12653 |
| Anti-rat IgG | 1:200 | Sigma Aldrich SAB3700596 |
| Anti-rabbit IgG | 1:200 | Bio-Rad 170-6515 |

**Supplementary Table 4:** Antibodies used for immunofluorescence

| **Antibody** | **Dilution** | **Reference** |
| --- | --- | --- |
| Anti-CD3 | 1:100 | Agilent GA50361-2 |
| Anti-CD206 | 1:100 | Abcam ab64693 |
| Anti-endomucin | 1:200 | Santa Cruz sc-65495 |
| Anti-Glut1 | 1:100 | Novus NB110-39113 |
| Anti-podoplanin | 1:100 | Sigma‒Aldrich MABT1512 |
| Anti-αSMA | 1:100 | Abcam ab5694 |
| Anti-rat IgG-Alexa Fluor (AF)488 | 1:200 | Thermo Fisher Scientific A11006 |
| Anti-syrian hamster IgG-AF488 | 1:200 | Thermo Fisher Scientific A21110 |
| Anti-rabbit IgG-AF568 | 1:200 | Thermo Fisher Scientific A10042 |
| Anti-rat IgG-AF568 | 1:200 | Thermo Fisher Scientific A11077 |

**Supplementary Table 5:** Primers for qRT‒PCR

| **Genes** | **Sequence (5’ to 3’)** | **Reference** |
| --- | --- | --- |
| *Actb* | TCTACAAATGTGGCTGAGGACT | - |
|  | GAGGGACTTCCTGTAACCACTT |  |
| *Cox2* | TGC TGG TGG AAA AAC CTC GT | - |
|  | AAA ACC CAC TTC GCC TCC AA |  |
| *Cxcl12* | CCA GAG CCA ACG TCA AGC AT | - |
|  | CAG CCG TGC AAC AAT CTG AA |  |
| *Ifng* | - | Bio-Rad  qMmuCID0006268 |
| *Il1b* | GCC TGT GTT TTC CTC CTT GC | - |
|  | TGC TGC CTA ATG TCC CCT TG |  |
| *Il2* | - | Bio-Rad  qMmuCID0015786 |
| *Il6* | GCC TGT GTT TTC CTC CTT GC | - |
|  | GCC TGT GTT TTC CTC CTT GC |  |
| *Il10* | - | Bio-Rad  qMmuCED0044967 |
| *Il12a* | - | Bio-Rad  qMmuCID001668 |
| *Tnf* | - | Bio-Rad  qMmuCED0004141 |
| *Rps13* | GATGCTAAATTCCGCCTGAT | - |
|  | TAGAGCAGAGGCTGTGGATG |  |

**Supplementary figure 1. Gating strategy for the selection of different cell populations.** The total leukocyte population was selected by selecting CD45^hi^ cells and excluding doublets. FSC/SSC gate selection was performed by comparing with peripheral blood leukocytes. The different cell populations were subsequently selected according to the markers and regions explained in the figure and in supplementary table 5.

**Supplementary figure 2. Continuous endoglin overexpression does not alter the immune profile in the peripheral blood. (A)** Percentages of CD8^+^ T cells (p=0,1633), **(B)** total T cells (p=0, 1633), **(C)** CD4^+^ T cells (p=0,2674), **(D)** CD4^+^/CD8^+^ lymphocyte maturation stage (p=0,0608), **(E)** CD4^-^/CD8^-^ lymphocyte maturation stage (p=0,2468), **(F)** NK cells (p=0,9152), **(G)** B lymphocytes (p=0,5426), **(H)** neutrophils and MDSCs (p=0,8735), and **(I)** monocytes (p=0,9676) of total leukocytes (CD45^+^) [n(WT)=5; n(*ENG^+^*)=6].

**Supplementary figure 3. Continuous endoglin overexpression alters the immune profile of subcutaneous tumors**. **(A)** Percentages of CD8^+^ T cells (p=0,1178), **(B)** total T cells (p=0,7032), **(C)** CD4^+^ T cells (p=0,9216), **(D)** CD4^-^/CD8^-^ lymphocyte maturation stage (p=0,8786), **(E)** CD4^+^/CD8^+^ lymphocyte maturation stage (p=0,3643), **(F)** NK cells (*p-value < 0,05), **(G)** B lymphocytes (p=0,0833), **(H)** neutrophils and MDSCs (p=0,8237), and **(I)** monocytes (p=0,2533) of total leukocytes (CD45^+^) [n(WT)=8; n(*ENG^+^*)=9].

**Supplementary figure 4. Expression of different cytokines present in the tumor microenvironment. (A)** qPCR analysis of the expression of antitumor cytokines, such as *Tnf* (p=0,7423), **(B)** *Ifng* (p=0,6419), **(C)** *Il2* (p=0,1719), and **(D)** *Il12a* (**p-value < 0,01) [n(WT)=8; n*(ENG^+^*)=8]. **(E)** qPCR analysis of protumor cytokines, such as *Il1b* (p=0,0572), **(F)** *Il10* (p=0,3240), **(G)** *Il6* (*p-value < 0,05), **(H)** *Cxcl12* (*p-value < 0,05), and **(I)** *Cox2* (**p-value < 0,01) [n(WT)=8; n*(ENG^+^*)=8].

**Supplementary figure 5. Characterization of tumor vascularization in lung tumors. (A)** Immunofluorescence of the pneumocyte marker Podoplanin (green) and the vessel marker Endomucin (red). In the co-option areas, cancer cells fill the alveolar air spaces (asterisk), preserving the alveolar walls and the associated alveolar capillaries (arrow). On the other hand, in the angiogenic area, we observed neovascularization (triangle) and destruction of the lung parenchyma (circle). **(B)** Angiogenic and co-option areas were analyzed with respect to the total tumor area. Endoglin overexpression does not determine the generation of a predominant angiogenic or co-option vascular profile [n(WT)=22 (from 5 mice); n(*ENG^+^*)=23 (from 5 mice), p=0,6677].

**Supplementary Figure 6. Immunofluorescence images with individual channels in the subcutaneous tumor model.** (A) Immunofluorescence staining of the vessel marker Endomucin (red), mural cell marker α SMA (green) and DAPI (blue) used to evaluate vascular density and mural coverage shown in Figure 1C. (B) Immunofluorescence staining of Glut1 (green) and DAPI (blue) corresponding to the hypoxic areas quantified in Figure 1F. (C) Immunofluorescence staining of CD206 (red) and DAPI (blue) illustrating M2 TAM infiltration shown in Figure 2C. (D) Immunofluorescence staining of the vessel marker Endomucin (red), mural cell marker α SMA (yellow), CD3⁺ lymphocytes (green) and DAPI (blue) corresponding to the analysis of perivascular CD3⁺ lymphocytes in Figure 3A. These images display all individual channels separately to facilitate visualization and interpretation of the markers used in the main figures.

**Supplementary Figure 7. Immunofluorescence images with individual channels in the lung tumor model.** (A) Immunofluorescence staining of the vessel marker Endomucin (red), mural cell marker α‑SMA (green) and DAPI (blue) used to evaluate vascular density and mural coverage shown in Figure 4C. (B) Immunofluorescence staining of Glut1 (green) and DAPI (blue) corresponding to the hypoxic areas quantified in Figure 4F. (C) Immunofluorescence staining of CD206 (red) and DAPI (blue) illustrating M2 TAM infiltration shown in Figure 5C. These images display all individual channels separately to facilitate visualization and interpretation of the markers used in the main figures.
